# Opportunities to improve the impact of two national clinical audit programmes: a theory-guided analysis

**DOI:** 10.1186/s43058-022-00275-5

**Published:** 2022-03-21

**Authors:** T. A. Willis, S. Wood, J. Brehaut, H. Colquhoun, B. Brown, F. Lorencatto, R. Foy

**Affiliations:** 1 Leeds Institute of Health Sciences, University of Leeds, Leeds, LS2 9JT UK; 2 Ottawa Hospital Research Institute, Ottawa, Canada; 3 School of Epidemiology and Public Health, University of Ottawa, Ottawa, Canada; 4 Department of Occupational Science and Occupational Therapy, University of Toronto, Toronto, Canada; 5 Centre for Primary Care and Health Services Research, University of Manchester, Manchester, UK; 6 Division of Informatics, Imaging and Data Science, University of Manchester, Manchester, UK; 7 Centre for Behaviour Change, University College London, London, UK

**Keywords:** CP-FIT, Audit and feedback, Clinical audit, Qualitative

## Abstract

**Background:**

Audit and feedback is widely used in healthcare improvement, with evidence of modest yet potentially important effects upon professional practice. There are approximately 60 national clinical audit programmes in the UK. These programmes often develop and adapt new ways of delivering feedback to optimise impacts on clinical practice. Two such programmes, the National Diabetes Audit (NDA) and the Trauma Audit Research Network (TARN), recently introduced changes to their delivery of feedback. We assessed the extent to which the design of these audit programmes and their recent changes were consistent with best practice according to the Clinical Performance Feedback Intervention Theory (CP-FIT). This comprehensive framework specifies how variables related to the feedback itself, the recipient, and the context operate via explanatory mechanisms to influence feedback success.

**Methods:**

We interviewed 19 individuals with interests in audit and feedback, including researchers, audit managers, healthcare staff, and patient and public representatives. This range of expert perspectives enabled a detailed exploration of feedback from the audit programmes. We structured interviews around the CP-FIT feedback cycle and its component processes (e.g. Data collection and analysis, Interaction). Our rapid analytic approach explored the extent to which both audits applied features consistent with CP-FIT.

**Results:**

Changes introduced by the audit programmes were consistent with CP-FIT. Specifically, the NDA’s increased frequency of feedback augmented existing strengths, such as automated processes (CP-FIT component: Data collection and analysis) and being a credible source of feedback (Acceptance). TARN’s new analytic tool allowed greater interactivity, enabling recipients to interrogate their data (Verification; Acceptance). We also identified scope for improvement in feedback cycles, such as targeting of feedback recipients (Interaction) and feedback complexity (Perception) for the NDA and specifying recommendations (Intention) and demonstrating impact (Clinical performance improvement) for TARN.

**Conclusions:**

The changes made by the two audit programmes appear consistent with suggested best practice, making clinical improvement more likely. However, observed weaknesses in the feedback cycle may limit the benefits of these changes. Applying CP-FIT via a rapid analysis approach helps identify strengths and remediable weaknesses in the design of audit programmes that can be shared with them in a timely manner.

**Supplementary Information:**

The online version contains supplementary material available at 10.1186/s43058-022-00275-5.

Contributions to the literature

- Audit programmes often make incremental changes to their methods to increase their impacts on clinical practice.
- This study demonstrates the practical application of Clinical Performance Feedback Intervention Theory (CP-FIT), using rapid analysis of expert interviews, to two national audit programmes which had recently introduced changes to their feedback methods.
- Such changes can augment feedback methods in ways that are consistent with best practices according to CP-FIT. However, their impacts may be limited by a range of remediable weaknesses in the feedback cycle.

## Background

Audit and feedback is widely used to improve the quality of healthcare internationally, including approximately 60 national clinical audit programmes in the United Kingdom (UK) [1]. It involves reviewing performance against explicit standards (audit) and presenting data to guide improvement (feedback). It is an intervention that can be delivered at scale and at a comparatively low cost, and a Cochrane review of 140 randomised trials showed it produced a median 4.3% absolute improvement in professional practice [2]. Knowledge of the optimal components of audit and feedback can help to make its effectiveness more consistent, with potentially substantial impacts upon clinical practice (in a quarter of trials, the improvement exceeded 16%).

Evidence-based methods to enhance audit and feedback are known, e.g. incorporating goals and action plans [2]. However, evolving theory in this field may also suggest ways of improving effectiveness. The majority of audit and feedback interventions in research studies do not explicitly draw upon theory [2, 3]. The extent to which the organisation and delivery of ‘real-life’ national audit programmes reflect best practice from theory is less known.

**Fig. 1:**
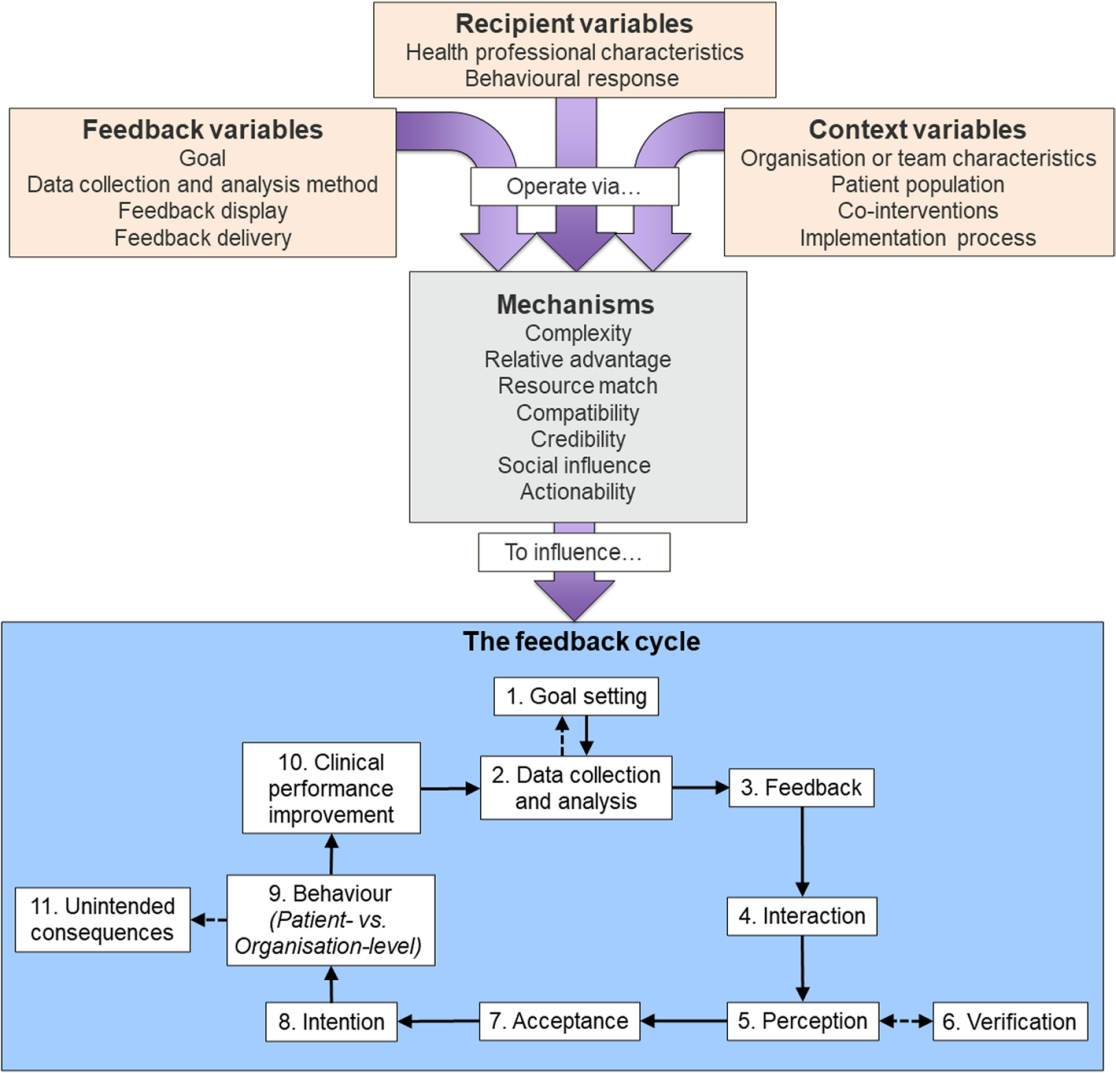
Clinical Performance Feedback Intervention Theory’s variables and explanatory mechanisms and their influence on the feedback cycle. Solid arrows are the necessary pathways for successful feedback; dotted arrows represent the potential pathways [4]

The Clinical Performance Feedback Intervention Theory (CP-FIT) offers the most comprehensive theory to date on the conditions for optimal audit and feedback [4]. It builds upon 30 existing behaviour change theories and frameworks, providing an integrated theory of feedback specifically relevant to healthcare. It proposes that effective audit and feedback is a cyclical process consisting of goal setting and audit, feedback message production, perception and Acceptance of feedback message, recipient Intention to respond, action (at the individual and organisational levels), and, ultimately, care quality improvement (Fig. 1). Progress through the cycle will be weakened, or halted entirely, if any individual component fails.

We applied CP-FIT to two UK national clinical audit programmes (summarised in Table 1), which recently introduced changes to their feedback delivery. The National Diabetes Audit (NDA) [5] increased the frequency of data release to primary care recipients from annual to quarterly intervals, consistent with a suggestion to provide feedback as soon as possible following data collection [6]. The Trauma Audit Research Network (TARN) [7] introduced a new system (‘TARN Analytics’) that offers users greater interactivity to explore data, consistent with a suggestion to provide short, actionable messages followed by optional detail [6].

**Table 1:** Description of the two national clinical audits

**National Diabetes Audit (NDA)** [5]	
The NDA Programme comprises four modules: the National Diabetes Core Audit, the National Pregnancy in Diabetes Audit, the National Diabetes Footcare Audit, and the National Inpatient Diabetes Audit. The NDA helps improve the quality of diabetes care by enabling participating NHS services and organisations to assess local practice against the NICE guidelines (e.g. the proportion of eligible patients with diabetes that achieve target levels of blood pressure, cholesterol, and blood sugar), compare their care and outcomes with similar services and organisations, identify gaps or shortfalls that are priorities for improvement, identify and share best practice, and provide comprehensive national pictures of diabetes care and outcomes in England and Wales. Audit reports provide national- and local-level information on, for example, prevalence, care process completion, and treatment target achievement. Our study focused on the quarterly release of data included in the Core Audit.	
**Trauma Audit Research Network (TARN)** [7]	
TARN is the National Clinical Audit for traumatic injury and is the largest European Trauma Registry, holding data on over 800,000 injured patients including over 50,000 injured children. TARN aims to measure and monitor the processes and outcomes of care (e.g. the proportion of patients with head injury receiving a CT scan within 60 min) to demonstrate the impact of trauma networks, providing local, regional, and national information on trauma patient outcomes, and thereby help clinicians and managers to improve trauma services. Individual patient data are inputted manually at the trauma unit to an online data collection and validation system, aiming to be available within 25 days of patient discharge or death. Our study focused on the online, ‘TARN Analytics’ tool: a reporting tool designed to offer users a more dynamic method of viewing and manipulating their data, e.g. by supporting the creation and sharing of data visualisations.	

We drew upon CP-FIT to apply a theoretical lens to evaluate two national audit programmes and hence identify their strengths and opportunities for improvement.

## Methods

### Design

We had originally designed a larger-scale evaluation of the two audit programmes, including observational research and interviews with service commissioners, clinicians, and managers. However, the coronavirus pandemic resulted in the suspension of all non-essential UK healthcare research. We therefore completed a pragmatic, non-intrusive evaluation, within the time limits of a funded research programme. We drew upon semi-structured, expert interviews, using CP-FIT as a framework. We used a rapid approach to analysis and interpretation. Rapid techniques within qualitative analysis are considered appropriate to provide valid findings and recommendations within a short timeframe [8–11]. Our report follows the COREQ recommendations for qualitative studies [12] (Additional file 1).

### Participants

We aimed for a sample of up to 20 individuals from our existing network of co-investigators, collaborators, and advisers, all of whom were directly connected to our National Institute for Health Research-funded study to optimise feedback from national clinical audits. This included audit and feedback researchers, national clinical audit leads and commissioners, clinicians targeted by national audits, and patient and public representatives. We approached individuals informed by their experience and knowledge in clinical audit research and practice, seeking representation across the various roles and backgrounds available.

### Theoretical framework: Clinical Performance Feedback Intervention Theory (CP-FIT) (Fig. 1 [4];)

CP-FIT offers a comprehensive explanation of how clinical feedback is believed to work and the factors that influence its effectiveness. This includes three categories of variables, relating to the feedback itself (e.g. how the feedback is displayed and delivered), the recipient (e.g. characteristics of the targeted health professionals), and the context (e.g. the existence of and interaction with other improvement interventions). These variables are proposed to operate via a set of seven explanatory mechanisms to influence the feedback cycle. For instance, ‘credibility’ refers to the feedback’s perceived trustworthiness and reliability; users are more likely to engage with credible feedback. CP-FIT also presents a set of evidence-based, ‘high-confidence’ hypotheses on how to enhance the effectiveness of feedback. For example, the ‘action planning’ hypothesis reads, ‘feedback interventions are more effective when they provide solutions to suboptimal performance (or support recipients to do so)’; the ‘ownership’ hypothesis proposes, ‘feedback interventions are more effective when recipients feel they “own” it, rather than it has been imposed on them.’

We used the eleven components from the CP-FIT feedback cycle as the framework for interviews and analysis [4]. These are outlined in Table 2, together with definitions and corresponding questions from the interview topic guide. Some interviews covered the entire cycle whilst others focused on specific components, depending upon participants’ background and expertise.

**Table 2:** The CP-FIT feedback cycle components and associated interview questions

CP-FIT feedback cycle component	Definition	Interview question
1. Goal setting	CP-FIT hypothesises that feedback is more effective when the clinical performance standards are considered important and relevant to recipients’ roles.	Are the standards of clinical performance clear?
2. Data collection and analysis	Automated data collection and analysis processes are generally recommended. Manual collection and analysis are often hindered by a lack of time or skills.	Who does the data collection?
3. Feedback	Current best evidence supports more frequent provision of feedback. Data should also be as recent as possible, which may enhance subsequent cycle components (Acceptance, Intention, and behaviour) and encourage identification of suboptimal performance. Other relevant factors include problem-solving and action planning, i.e. helping recipients identify and introduce solutions to improve.	What feedback is communicated?
4. Interaction	This component includes the method of delivery and how recipients interact with the feedback, e.g. is it delivered directly to clinicians or do they need to seek it out?	How is the feedback received?
5. Perception	Feedback is more effective when it is user-friendly. Provision of a comparator (e.g. showing performance benchmarked against appropriate others) is considered to facilitate the perception, Intention, and behaviour components.	How is the feedback understood?
6. Verification	A potential component between perception and Acceptance where, if the feedback permits, recipients can explore the data underlying performance.	Can the recipients interrogate the data?
7. Acceptance	Acceptance is facilitated when recipients believe the feedback presents a true representation of their performance. Users are more likely to engage with credible feedback, which facilitates several cycle components.	Is there Acceptance of the feedback?
8. Intention	Ideally, recipients form Intentions to take actions to improve performance in response to the feedback.	Does the feedback elicit a planned response?
9. Behaviour	Feedback that has been received, understood, and accepted will ideally be followed by a planned behavioural response. A distinction is made between patient-level responses, i.e. relating to the care of individuals, and those at the organisational-level with impacts across the wider healthcare system.	Is the behavioural response at patient or organisation level?
10. Clinical performance improvement	Organisation-level behaviours are associated with greater clinical performance improvement potential as they enable multiple patient-led behaviours by enhancing the clinical environment in which they occur.	Are there positive changes to patient care as a result of feedback?
11. Unintended consequences	CP-FIT acknowledges the potential for both positive and negative unintended outcomes of feedback interventions. Examples include improved record-keeping, or manipulation of patient populations to artificially improve performance, respectively.	Are there any unintended consequences as a result of the feedback?

### Procedure

Interviews were completed by SW, lasted approximately 30–45 min, and were conducted remotely using telephone or video links. We first interviewed senior individuals from NDA and TARN to establish an understanding of how their programmes currently work, how their data are collected and processed (i.e. the ‘audit’ of audit and feedback), how their feedback is delivered to recipients, and clarify their recent innovations. These interviews helped us to develop short summaries of both audits that were provided to subsequent participants not already familiar with the audits (Additional file 2). We also sent participants an outline of CP-FIT, with linked interview questions, to familiarise them with the theory and help to focus attention on individual cycle components (Additional file 3).

We invited participants to choose to discuss one or both audits. We initially used open-ended prompts to encourage discussion about features of the feedback cycle of particular interest. In later interviews, we ensured that specific cycle components were considered if they had not been covered in earlier interviews. Field notes were made immediately after each interview. Audio recordings were transcribed verbatim.

### Analysis

We conducted a rapid, structured deductive content analysis of interviews exploring outputs from two national clinical audits. Our approach drew upon existing techniques [8, 9] and followed a two-stage process. We managed the data using Microsoft Excel. First, rather than line-by-line coding, individual transcripts were summarised using a one-page template that used the CP-FIT feedback cycle as a framework. Data were deductively added to the template to summarise participants’ thoughts about how the cycle components applied to the audit(s). The template contained space for recording other issues that emerged inductively from the data and fell outside of the components specified in the CP-FIT feedback cycle. Most frequently, this concerned points raised in relation to particular clinical audits other than NDA or TARN, and therefore outside of the focus of our study.

Templates were populated with key points and illustrative quotes. To ensure the reliability of interpretation, SW and TW independently prepared one-page summaries from the same two transcripts (one each focusing on NDA and TARN). These were checked to ensure that similar statements were generated within the different CP-FIT cycle components. Following discussion, two further transcripts were summarised and compared, with differences resolved by discussion. The remaining summaries were divided between SW and TW and completed individually, with regular meetings to check agreement. Examples of interview summaries are provided in Additional files 4 and 5. In the second stage, individual summaries were combined to create a matrix, one per audit, populated with summaries of participant comments that mapped to the cycle components. Each matrix was divided into two sections, the first including positive comments and the second detailing weaknesses and potentially failed components. The data for each audit (NDA and TARN) were considered separately; we did not combine data across audits. Data were analysed alongside CP-FIT’s high-confidence hypotheses. Where data linked to a particular hypothesis, it was noted whether the associated audit programme had achieved the hypothesis or not.

SW and TW met to identify and agree key strengths and weaknesses for each audit in relation to CP-FIT. We set ourselves a goal to identify three of each for each audit to ensure that we delivered practical and focussed outputs for our collaborating audit programmes. We shared our findings with senior managers at NDA and TARN whilst finalising the manuscript. We were particularly keen to ensure that the potential limitations identified for both audits were justified.

Data were analysed in relation to all components of the feedback cycle, for both audits. However, to reduce unnecessary words and allow greater focus upon the most important findings, the study team identified which cycle components to present here, and which to include in Additional file 6. All authors contributed to this decision process.

### Ethical approval

The study was approved by the University of Leeds School of Medicine Research Ethics Committee (ref: MREC 18-051) and the Health Research Authority (IRAS ID: 258139).

## Results

We invited 25 individuals to participate. Nineteen responded before our recruitment deadline, and we completed eighteen interviews, one involving two people, between June and August 2020. Eight participants were clinicians or managers, seven researchers, four patient and public representatives, and three audit providers; several interviewees had multiple roles (Table 3).

**Table 3:** Participant demographic information, role, and audit discussed

Participant characteristics	Number	Audit discussed	
		National Diabetes Audit	Trauma Audit Research Network
Role			
Audit and feedback researcher	4	2	4
Audit and feedback researcher and general practitioner	3	3	1
Audit provider	3	1	2
Patient and public representative	4	3	2
Hospital consultant	2	2	2
Hospital consultant and audit lead	2	1	1
Major trauma network manager	1	–	1
Total^a^	19	12	13
Location of participant			
Canada	1		
England	17		
Scotland	1		
Total	19		
Sex			
Male	11		
Female	8		
Total^b^	19		

^a^Several participants discussed both audits and thus the total values are greater than the number of interviews

^b^One interview comprised two people (one male, one female)

Table 4 presents an overview of the extent to which both audit programmes were considered to have achieved the CP-FIT feedback cycle. Cells indicating mixed features (+/-) indicate where interviewees identified both strengthening and limiting features of the feedback. We present our findings first for NDA, then TARN, focusing on the components considered most relevant when identifying three strengths and three areas for improvement for each audit. We made this pragmatic decision in setting ourselves goals to deliver key ‘headline’ measures for our national audit programme partners. The results for the remaining feedback cycle components are provided in Additional file 6.

**Table 4:** Summary of the extent to which CP-FIT feedback cycle components are considered to have been achieved by both audit programmes

	Feedback cycle process	National Diabetes Audit	Trauma Audit Research Network
1	Goal setting	++	+
2	Data collection and analysis	++	+/-
3	Feedback	+	+
4	Interaction	-	+/-
5	Perception	-	+/-
6	Verification	+/-	++
7	Acceptance	+	+
8	Intention	-	-
9	Behaviour	-	-
10	Clinical performance improvement	-	-
11	Unintended consequences	n/a	n/a

++, component strongly present or achieved; +, component present or achieved; -, component absent; +/-, mixed features identified

### National Diabetes Audit

#### Data collection and analysis (Fig. 1, feedback cycle component 2)

Automated data collection places no additional demands on practice staff. This reduces the *complexity* [italics indicate CP-FIT explanatory mechanisms] of participation, increasing the likelihood of progress through the feedback cycle. The CP-FIT ‘ownership’ hypothesis is also relevant here as the lack of staff involvement in collecting data may undermine ownership.

#### Feedback

Interviews focused particularly on the move to quarterly data release, introduced partly in response to user requests for quicker access to performance data. Participants supported the initiative: more frequent feedback would *help people to get on top of their data* (D9, hospital consultant and audit lead), and [It] *is good as lots can change in a year and you wouldn’t know if you were improving* (D17, audit and feedback researcher and GP). One participant valued an audit’s ability to be *reactive* and respond quickly to issues emerging from the data, or to topical health questions, e.g. the impact of COVID-19 upon specific clinical indicators. She questioned whether the NDA could do this, describing the audit as *monolithic* (D14, PPI representative and former national audit developer).

However, more frequent feedback may cause *alert fatigue* (D18, audit and feedback researcher), and its impact depends on the number of patients included in the feedback, and the time taken for any change to occur.

#### Interaction

NDA feedback may often fail to reach its intended audience. Participants with primary care roles reported having little awareness of the feedback, which was not discussed by their practice teams. It had to compete for attention with other priorities:

*“As a recipient, I think that’s probably been one of the key issues is that I’ve just not looked at it very much because it’s not really been pushed in my face or kind of promoted”* D16, audit and feedback researcher and GP

This critical break in the feedback cycle means that feedback content cannot be received and acted upon.

#### Perception

Interviewees expressed major reservations about the presentation of feedback data, variously described as *very dense* (D7, hospital consultant), *overwhelming* (D11, audit and feedback researcher), and *really quite dysfunctional and unappealing* (D9, hospital consultant). Participants consistently observed that the data were practically impenetrable on first look, with few users likely to have the time nor inclination to extract what was personally relevant.

*“*[Quarterly feedback]* is useful as long as it is decipherable data. If you are just sent a massive spreadsheet that you don’t read, it doesn’t really matter whether it is quarterly or annually, it’s still not going to be read.”* D7, hospital consultant

One interviewee highlighted the cognitive effort required to act on the feedback:

*“*[Recipients] *have to do a lot of work in order to take their one line from this data…and turn it into useful information. …. Unless it’s made dead easy, and people are interested and want to engage in it then they won’t even make use of the easy to use displays, never mind the stuff where they actually have to go and do some work.”* D18, audit and feedback researcher

Another factor that hindered the interpretation of feedback was the lack of a comparator to help recipients understand their performance against others or over time. Whilst the NDA data release permits users to compare performance against others, it requires manipulation of a complex spreadsheet.

*“If I find that I’m 20% lower than where other people are just now that sets off alarm bells. … On the other hand, if I’m doing 20%, 30% better than expected, actually that is really useful to know… I can say, ok, so that’s something I’m not going to worry about for the next two-three months.”* D2, audit and feedback researcher and GP

*“The numbers alone are not enough. They need to be accompanied by specific targets so that progress towards these can be assessed.”* D13, PPI representative

#### Acceptance

Participants believed that the feedback, if read, would be accepted by recipients. The NDA was considered credible, and the clinical content was recognised as important. One GP participant noted that there was no segmentation of the data. Therefore, suboptimal performance may be attributed to, for example, patient demographics rather than the functioning of the practice team. Consequently, progress through the cycle to Intention, behaviour, and performance improvement would be unlikely.

#### Intention and behaviour

These two components were typically discussed together. One interviewee believed that the feedback would prompt recipients to consider their performance and ask themselves how they might act in response:

*“It all has a good story to tell and I think that good story enables clinicians and managers to apply themselves to, ‘this is how it works locally, this is what we’ve got, what are we doing about it?’”* D13, PPI representative with experience of CCG management

The feedback generally related to regular clinical behaviours that did not require new learning or training. However, several interviewees identified a problem in that it is not made explicitly clear *what* is required of recipients in response to the feedback, beyond a general call to ‘do better’:

*“I don’t know what response they want to elicit, other than work harder?”* D2, audit and feedback researcher and GP

*“You want to have ideally an action to, for someone to at least think about, and it’s not clear to me … that there’s anything that they should be thinking about doing”* D18, audit and feedback researcher

Participants indicated that the feedback could be enhanced by incorporating recommendations on how to improve, which is consistent with two CP-FIT hypotheses: that feedback is more effective when it helps recipients identify and develop solutions to reasons for suboptimal performance [‘problem solving’], and provides solutions to suboptimal performance [‘action planning’]. Providing clear guidance would reduce the effort required of recipients in identifying solutions, thereby reducing feedback *complexity* and making it more *actionable*. One suggestion was to accompany the feedback with tools that support the creation of relevant patient lists so that they might be invited for review:

*“They don’t provide patient lists so I think that’s probably, you know, that’s a major downfall… you need patient-level change as well and patient-level change really only happens if you have those patient lists.”* D16, audit and feedback researcher and GP

The absence of recommendations may undermine any potential gains arising from more frequent data release:

*“It’s not just a matter of having the data and having it good quality, and giving it to the practitioners, you have to do more than that in order to get them to use it.”* D18, audit and feedback researcher

We identified three main strengths of the NDA feedback that were consistent with CP-FIT and three opportunities for improvement (Table 5).

**Table 5:** NDA feedback: key strengths likely to facilitate successful progress through the CP-FIT feedback cycle and opportunities for improvement. Associated feedback cycle components are displayed in brackets

Strengths	Opportunities for improvement
More frequent data release This appears to meet user needs and is consistent with the best evidence about improving feedback effectiveness (Acceptance, Intention).	Delivery to target recipients The feedback may be failing to reach much of its intended audience. If staff are unaware of the feedback then it cannot be discussed nor prompt improvement (Interaction).
Automated data collection This minimises the impact upon the staff and helps to ensure a large, accurate dataset. It also reduces complexity and strengthens the initial processes of the cycle (Data collection and analysis).	Presentation Those who do receive it may not read it: participants found it off-putting and impenetrable (Perception).
Respected source and widely accepted indicators The feedback is considered to come from a credible source; the indicators are recognised as relevant and important (Acceptance).	Interpretation Too much was required of users to produce useful comparator detail (Perception, Intention).

### Trauma Audit Research Network

#### Data collection and analysis (Fig. 1, feedback cycle component 2)

TARN data are manually processed. This can be time-consuming and requires expertise to ensure accuracy. Two participants with experience in other secondary care audit programmes identified manual data collection as a potential weakness. One explained that manual processes were associated with ongoing questions about whether all data were necessary, who completes the work, and how this is resourced. Conversely, manual processing may heighten feelings of ‘ownership’. Participants perceived TARN data collection to be efficient and rigorous, but saw value in introducing automation:

*“We should be moving to a world in which the data is collected not essentially paper-based, manually, but is collected more remotely, electronically, efficiently… I think there’s a lot of scope for doing that and I think that’s something important.”* D12, hospital consultant

#### Interaction

Descriptions of the TARN Analytics tool were generally positive, but this did not guarantee use. Recipients may take time to adjust to new systems, with some preferring to use their own trusted methods. The trauma network manager described regularly checking TARN data but did not actually use the new system:

*“I didn’t have a motivation to go in and teach myself how to do it… I’ve probably been in *[the new system]* in all truth probably less than half a dozen times between *[getting access]* and now because I keep going back to the old ways of working.”* D5, trauma network manager

A participant with experience of developing a national audit acknowledged TARN Analytics’ impressive functionality but questioned how widely it might be used:

*“I guess it would be interesting to know how well it was being used, or if it was being used. Because I know in *[another audit]* there were some very active units who were always requesting data and always wanting information. But there were other units who never, you know, they submitted data, but the staff, there wasn’t a culture, really, of using information to examine and evaluate their practice.”* D14, PPI representative and former national audit developer

Another issue concerned the feedback’s intended audience. Several participants questioned whether there might be better signposting for different groups of users. For example:

*“I guess this appeals to people like me and will appeal to medical directors…and it probably will be important in terms of influencing doctors perhaps and some nurses, but not everybody… there’s definitely a thing for me about the difference in audiences. Whose performance you are actually trying to, whose behaviour are you trying to change?”* D9, hospital consultant

#### Perception

The TARN Analytics dashboard was considered attractive and user-friendly, facilitating progress through the feedback cycle. However, there remained scope for improving design and reducing complexity:

*“What it lacks is sort of headlines, labels to orient the viewer. *[Presenting similar information in different ways]* is adding to the overall sort of cognitive load of having to unpack it. I think that there’s a trade-off between in general you want to present things in terms of words and graphs, present them in multiple ways, but there I think this probably goes a bit too far.”* D18, audit and feedback researcher

TARN feedback presents performance against the national median. One participant questioned the effectiveness of this comparator; for them, a higher target would be more useful:

*“*[Recipients] *have got to understand the national average…and whether that is good or bad, and obviously that doesn’t come across in a doughnut or in a graph, it’s just the national average. … *[Hospital X]* is probably our lowest one in some areas and they are still above national average. We obviously don’t want them to focus and think, ‘well, we’re above national average, brilliant!’ We have to repeatedly say, ‘look, you’re far worse than *[Hospital Y]* so you’d better be trying to get better… no one should be aspiring to be average, everyone should be aspiring to upper quartile. … I would if it was me delete all average figures and only have where the upper quartile figure is.”* D5, trauma network manager

#### Verification

Participants recognised that TARN Analytics enhanced users’ ability to interrogate their data, strengthening the Verification component of the cycle. Those familiar with other audits described the ability to ‘drill down’ into data as something regularly requested by colleagues:

*“I think that’s a good idea, I mean that’s almost the comment that comes back on *[our]* audit is that sites want to drill down their own data, they want to understand their own practice in more detail.”* D12, hospital consultant

Providing optional further detail for those who wish to explore it is consistent with recommended practice. One participant referred to *levels of interest* and catering for different types of users:

*“You have a sort of vaguely interested person and then you have a person who is more interested, and then you have an expert, and you provide sort of different levels of information”* D19, hospital consultant and national audit lead

The participant working in trauma care suggested that the new tool would have time-saving benefits. He described an occasion when achievement on an indicator had suddenly and unexpectedly declined. The staff spent a long time investigating the issue and what might explain the change. He believed that TARN Analytics would simplify such processes:

*“I would hope it could help pinpoint faster that it is, e.g. two outliers that have caused it, or is it one month of the year that has caused it, because otherwise you waste a lot of time analysing, reanalysing everything to find the contributing factor that has changed the number.”* D5, trauma network manager

#### Acceptance

It was apparent that TARN feedback was respected and considered credible by both its target audience and clinicians from other specialties. The trauma network manager (D5) confirmed that the data were an integral part of care at all levels of his organisation. Data were used by teams to assess the impact of quality improvement projects and featured in peer review visits and local executive meetings.

#### Intention and behaviour

Participants again noted that it was not made clear what was required of recipients in response to feedback. The trauma network manager (D5) suggested that action was required primarily of clinicians:

*“For me, the target audience is less people like me and more actually clinical, clinicians and surgeons and consultants.”*

He appeared to consider himself more as an intermediary who could extract detail from the feedback and share it with others, e.g. directors and clinicians. It was not clear whether or how such individuals would then act.

One participant discussed how feedback should be targeted, such that specific recommendations are congruent with recipients’ organisational role. For example, if high-level actions are required, then any recommendations need to be for tasks that appropriate individuals or teams can actually deliver. Moreover, recipients at those levels must also understand that action is required of them.

*“We’re trying to influence lots of different people and if we were trying to do that all with data, they might all need to see perhaps a slightly different version, or at least a version that would ideally suit them.”* D9, hospital consultant and national audit lead

#### Clinical performance improvement

The trauma network manager stated that TARN feedback had *transformed care*. This was not supported by specific examples, unfortunately. Other participants felt that TARN could do more to demonstrate genuine impact, with one interviewee considering this a common weakness across audit programmes. She had searched the TARN website for examples of performance improvement attributable to TARN, without success:

*“Where are the tangible benefits about change in practice, about how this information actually benefitted patients? … not enough about how the database was exploited for patient benefit… there is a lot about feeding back to organisations… but I couldn’t grasp… why would you want to continue funding this database, what changes, you know, are in evidence?”* D14, PPI representative and former national audit developer

We again identified three strengths of the feedback and three opportunities for improvement as a practical output for our audit partners (Table 6). The strengths facilitate achievement of the cycle components and encourage progress through the cycle. Opportunities for improvement concern issues most likely to inhibit progression. The results suggest relatively smooth progress around the feedback cycle up to Intention (component 7, Fig. 1), becoming more uncertain beyond that point.

**Table 6:** TARN feedback: key strengths likely to facilitate successful progress through the CP-FIT feedback cycle, and opportunities for improvement. Associated feedback cycle components are displayed in brackets

Facilitator	Opportunities for improvement
Enhanced interactivity The TARN Analytics tool was considered a useful innovation. It aligns with recommended practice, allowing users to ‘drill down’ into the data (Perception, Verification).	Action planning Providing recommended actions to guide recipients on how to improve would make the feedback more actionable (Intention, Behaviour).
Use of comparator Performance presented as relative to the national average, stimulating social influence (Perception, Intention).	Comparator flexibility Offering users the ability to select preferred comparator(s) could be more beneficial than a standard national average (Perception, Intention).
Respected source Feedback considered to come from a credible source. TARN recognised as an exemplar from which other audits could learn (Acceptance)	Evidence of impact Clearer demonstration of impact upon patient care would likely strengthen the feedback cycle (all).

## Discussion

This is the first study to apply a comprehensive, healthcare-specific theory of feedback (CP-FIT) [4] to assess the clinical audit programmes. Changes introduced by two audit programmes: increased frequency of feedback by the NDA and an analytics tool allowing interrogation and verification of feedback data by TARN, were consistent with best practices according to CP-FIT and augmented existing strengths. We also identified potential weaknesses in feedback cycles, such as the omission of specific actions plans for improvement and limited demonstration of audit impacts on patient care and outcomes. We have previously demonstrated variations in how feedback is delivered by UK national clinical audit programmes [13]. This closer analysis and comparison of two audit programmes has highlighted a range of opportunities to improve impacts specific to each programme.

Participants identified elements from both audits that were likely to facilitate progress through the CP-FIT feedback cycle and make it more likely that the audit programme would contribute to improved clinical performance. The NDA was described as an established, clinically relevant initiative, with efficient and widely respected data collection and analysis processes. The move to more frequent data release was consistent with the best evidence and consolidated existing programme strengths. TARN was recognised as a respected and pro-active audit programme, including by those from other secondary care specialities. The new TARN Analytics tool was described as an attractive and engaging feature and being of particular benefit to those users seeking to explore their data in more detail (augmenting the Perception, Verification, and Acceptance components of the feedback cycle).

Our findings highlight the importance of paying attention to all aspects of the feedback cycle to optimise impact. The feedback cycle is perhaps only as strong as its weakest link; any breakdown at one or more points in the cycle undermines the ability of an audit programme to drive improvement. For example, releasing feedback data more frequently is unlikely to enhance effectiveness if feedback has a limited or unknown reach and is difficult to comprehend.

The potential limitations we observed in relation to NDA and TARN are consistent with the wider audit and feedback literature. For example, ensuring that feedback is user-friendly and enables recipients to efficiently obtain the information they need (i.e. reducing cognitive load and feedback complexity) is considered a key requirement of an effective feedback intervention [4, 6]. User-centred design processes may help to ensure user-friendly feedback designs by identifying and rectifying problems earlier in the design process [14]. Similarly, feedback that also supports action planning or presents specific recommendations for action is consistently recommended by reviews in this field [2, 4, 6]. Ivers et al. [15] observed that recipients of an audit and feedback intervention to improve chronic disease management struggled to use it as a means to set specific improvement goals, highlighting the necessity of supporting this process as part of the intervention. CP-FIT hypothesises that offering solutions helps to facilitate the Intention and behaviour components of the feedback cycle. This is because providing solutions makes it simpler for health professionals to undertake each cycle component (i.e. reduces complexity) and makes it easier for them to act in response to the feedback (i.e. increases actionability).

Issues around the selection of comparators were identified in relation to both audits considered here. For the users of TARN, performance is routinely presented in comparison with the national average, whereas one interviewee commented that this might not be the most effective approach. A review of the comparators utilised in 146 randomised trials of audit and feedback [16] concluded that theory and evidence were underused in deciding which comparator(s) to include. One recommendation was that the comparator be tailored to individual recipients rather than benchmarking against the average as standard. This point was raised by our interviewee who described the importance of considering suboptimal performance from a local context and not the national perspective.

It is important to optimise major audit programmes, given upfront investments in establishing infrastructures and involving staff in data collection and the potential for far-reaching, population-level benefits [1]. A repeated analysis of UK national clinical audit reports identified trends towards the use of more effective feedback methods (e.g. greater use of recommendations for action) and identified further opportunities for improvement [13]. CP-FIT offers a rigorously derived framework for evaluating feedback interventions and explaining their observed or predicted effects [4]. Our rapid, framework-driven analysis demonstrates an approach to identify the strengths and limitations of audit programmes, and hence guide their further development.

We acknowledge the four main study limitations. First, our rapid evaluation, albeit as a necessary response to the coronavirus pandemic, had less rigour and depth compared to other qualitative methods. Nevertheless, rapid approaches to qualitative analysis are gaining recognition as an acceptable methodology within limited timeframes and have been successfully applied to other contexts [10]. Comparative studies have demonstrated that rapid analysis techniques can generate similar findings to in-depth approaches [8, 9, 11]. In particular, they can allow researchers to work productively with partners, such as audit programmes, that often seek timely and actionable recommendations.

Second, pandemic restrictions prevented the recruitment of actual feedback recipients as originally intended. The people we interviewed about TARN included three clinicians already familiar with the NDA but only a single individual with direct experience of TARN. Consequently, this individual’s perceptions feature prominently, although balanced with those of other secondary care clinicians. A related limitation is that we deliberately approached several expert participants with interests in audit and feedback whose perspectives will differ from more typical feedback recipients. Repeating this work with a sample of non-expert, end-users would offer the opportunity to compare and contrast the present findings.

Third, we did not formally assess for data saturation and cannot make claims to saturation of themes. However, our sample comprised 19 individuals offering a variety of practice, patient, and research perspectives on feedback. Time limitations prevented us from adding interviews beyond this point.

Fourth, our assessments considered the design and delivery of audit programmes in relation to theoretical best practices. However, what is theoretically recommended may not translate into real benefits when effectiveness is empirically evaluated [17]. We acknowledge the need to continue building the evidence base for audit and feedback [18].

Our findings have direct implications for both evaluated audit programmes: we have identified theory-based issues for both that may limit the effectiveness of both audits and their ability to improve patient care. Moreover, it is likely that the strengths and improvement opportunities identified are common to other national clinical audits. For example, difficulty in reaching those most able to respond effectively to feedback has been identified as a challenge for another national audit programme [19]. Audit programmes need to work through how to identify or develop their recipient communities and ensure that feedback is designed with consideration of their informational needs and capacity for effective action. We also noted a relative paucity of specific recommendations on how to improve performance. The Cochrane review of audit and feedback found that feedback was more effective when accompanied by clear targets and action plans [2]. Further research may examine relationships between key features of audit and feedback interventions, as delineated by CP-FIT, and their effects on clinical practice and outcomes. A further finding of wider relevance to audit programmes is the need to demonstrate their impact, both to improve recipient engagement and justify continued programme funding. Our findings may guide the external review and commissioning of national audit programmes.

## Conclusions

We have identified features of feedback from two national audit programmes likely to enhance and impede progress around the feedback cycle. For this commonly used improvement method, our findings suggest considerable scope to improve impacts on service delivery and patient care through these and other audit programmes. Those responsible for commissioning and delivering major audit programmes should encourage the systematic identification of key opportunities for improvement across the whole audit and feedback cycle.

## Supplementary Information

**Additional file 1.** COREQ checklist.

**Additional file 2.** Short summaries of NDA and TARN shared with participants prior to interview.

**Additional file 3.** CP-FIT feedback cycle and questions for discussion.

**Additional file 4.** Example of an interview summary (NDA).

**Additional file 5.** Example of an interview summary (TARN).

**Additional file 6.** Results for the remaining CP-FIT feedback cycle components.

## Data Availability

The datasets used during the current study are available from the corresponding author on reasonable request.

## Abbreviations

CCG: Clinical commissioning group
CP-FIT: Clinical Performance Feedback Intervention Theory
COREQ: Consolidated criteria for reporting qualitative research
GP: General practitioner
NDA: National Diabetes Audit
NHS: National Health Service
NICE: National Institute for Health and Care Excellence
PPI: Patient and public involvement
TARN: Trauma Audit Research Network
UK: United Kingdom
