# Supplementary material for: Opportunities to improve the impact of two national clinical audit programmes: a theory-guided analysis
Source: Implement Sci Commun. 2022 Mar 21;3:32. doi: 10.1186/s43058-022-00275-5 (PMC8935621; doi:10.1186/s43058-022-00275-5)
Supplement: Supplementary file 1 — Additional file 1. COREQ checklist. [file 43058_2022_275_MOESM1_ESM.docx]

**Additional File 1.**

**Consolidated criteria for reporting qualitative studies (COREQ): 32-item checklist**

| **No** | **Item and guide questions/descriptions** |  | **Location in manuscript (Section, page no)** |
| --- | --- | --- | --- |
| **Domain 1: Research team and reflexivity** | | |  |
| **Personal characteristics** | | |  |
| **1** | Interviewer/ facilitator  Which author/s conducted the interviews? | SW | Procedure, p7 |
| **2** | Credentials  What were the researcher’s credentials? E.g. PhD, MD | PhD | - |
| **3** | Occupation  What was their occupation at the time of the study? | Pharmacist researcher | - |
| **4** | Gender  Was the researcher male or female? | Female | - |
| **5** | Experience and training  What experience or training did the researcher have? | Qualitative and primary care experience | - |
| **Relationship with participants** | | |  |
| **6** | Relationship established  Was a relationship established prior to study commencement? | Yes | Participants, p7; Procedure, p8 |
| **7** | Participant knowledge of the interviewer  What did the participants know about the researcher? (e.g. personal goals, reasons for doing the research) | All participants were familiar with the research team, although not necessarily with the interviewer, and were approached because of the existing relationship. All were provided with a study information sheet prior to consenting for interview. | - |
| **8** | Interviewer characteristics  What characteristics were reported about the interviewer? (e.g. bias, assumptions, reasons and interests in the research topic) | No interviewer-related biases were identified. | - |
| **Domain 2: study design** | | |  |
| **Theoretical framework** | | |  |
| **9** | Methodological orientation and Theory  What methodological orientation was stated to underpin the study? | The study used CP-FIT as a framework for the semi-structured topic guide and coding of data. | Theoretical framework, p8 |
| **Participant selection** | | |  |
| **10** | Sampling  How were participants selected? (e.g. purposive, convenience, consecutive, snowball) | We targeted members of our existing network of research collaborators and advisers. | Participants, p7 |
| **11** | Method of approach  How were participants approached? (e.g. face-to-face, telephone, mail, email) | Email | - |
| **12** | Sample size  How many participants were in the study? | 19 | Results, p10 |
| **13** | Non-participation  How many people refused to participate or dropped out? Reasons? | Four individuals did not respond to the invitation to participate. A further two responded after the deadline for recruitment and were not recruited. | Results, p10 |
| **Setting** | | |  |
| **14** | Setting of data collection  Where was the data collected? E.g. home, clinic, workplace | Interviews were completed remotely via MicrosoftTeams or Zoom. | Procedure, p8 |
| **15** | Presence of non-participants  Was anyone else present besides the participants and researchers? | No | - |
| **16** | Description of sample  What are the important characteristics of the sample? (e.g. demographic data, date) | Interviews were completed June-August 2020. There were 18 interviews, one involved two people. Eight participants were clinicians or managers, seven researchers, four patient and public representatives and three audit providers (although interviewees could have multiple roles). | Results, p10; Table 1 |
| **Data collection** | | |  |
| **17** | Interview guide  Were questions, prompts, guides provided by the authors? Was it pilot tested? | Interviews were semi-structured around the processes in the feedback cycle. We provided participants with an overview of CP-FIT and summaries of the two audits ahead of interview. | Procedure, p8; Additional Files 2 & 3 |
| **18** | Repeat interviews  Were repeat interviews carried out? If yes, how many? | No | - |
| **19** | Audio/visual recording  Did the research use audio or visual recording to collect the data? | Audio recordings were made using a digital recorder. | Procedure, p8 |
| **20** | Field notes  Were field notes made during and/or after the interview? | Field notes were made immediately after each interview. | Procedure, p8 |
| **21** | Duration  What was the duration of the interviews? | 30-45 minutes | Procedure, p8 |
| **22** | Data saturation  Was data saturation discussed? | No | Discussion, p19 |
| **23** | Transcripts returned  Were transcripts returned to participants for comment and/or correction? | No | - |
| **Domain 3: analysis and findings** | | |  |
| **Data analysis** | | |  |
| **24** | Number of data coders  How many data coders coded the data? | Two: SW and TW | Analysis, p8-9 |
| **25** | Description of the coding tree  Did authors provide a description of the coding tree? | Interviews were coded using the CP-FIT feedback cycle as a framework. | Theoretical framework, p7; Analysis, p8-9 |
| **26** | Derivation of themes  Were themes identified in advance or derived from the data? | Data were coded against the feedback cycle to assess the extent to which each process was achieved. We also allowed for other issues to emerge inductively from the data. | Analysis, p8-9 |
| **27** | Software  What software was used to manage the data? | Microsoft Excel | Analysis, p9 |
| **28** | Participant checking  Did participants provide feedback on the findings? | No | - |
| **Reporting** | | |  |
| **29** | Quotations presented  Were participant quotations presented to illustrate the themes / findings? Was each quotation identified? (e.g. participant number) | Illustrative quotations are provided in the text, and identified by the participant number and their primary role. | Results, p10-19 |
| **30** | Data and findings consistent  Was there consistency between the data presented and the findings? | Yes | Results and Discussion |
| **31** | Clarity of major themes  Were major themes clearly presented in the findings? | Yes | Results and Discussion |
| **32** | Clarity of minor themes  Is there a description of diverse cases or discussion of minor themes? | No minor themes were identified. | - |
