# Supplementary material for: Opportunities to improve the impact of two national clinical audit programmes: a theory-guided analysis
Source: Implement Sci Commun. 2022 Mar 21;3:32. doi: 10.1186/s43058-022-00275-5 (PMC8935621; doi:10.1186/s43058-022-00275-5)
Supplement: Supplementary file 2 — Additional file 2. Short summaries of NDA and TARN shared with participants prior to interview. [file 43058_2022_275_MOESM2_ESM.docx]

**Additional File 2. Summaries of NDA and TARN shared with participants prior to interview.**

**The National Diabetes Audit (NDA) Summary**

<https://digital.nhs.uk/data-and-information/clinical-audits-and-registries/national-diabetes-audit>

<https://www.diabetes.org.uk/professionals/resources/national-diabetes-audit>

The National Diabetes Audit (NDA) is part of the National Clinical Audit and Patient Outcomes Programme (NCAPOP) which is commissioned by the Healthcare Quality Improvement Partnership (HQIP) and funded by NHS England. The NDA is managed by NHS Digital in partnership with Diabetes UK.

The NDA measures the effectiveness of diabetes healthcare against NICE Clinical Guidelines and NICE Quality Standards, in England and Wales.

**Aims of the core NDA:** to answers five questions:

- Is everyone with diabetes diagnosed and recorded on a practice diabetes register?
- What percentage of people registered with diabetes received the nine National Institute of Health and Care Excellence (NICE) key processes of diabetes care?
- What percentage of people registered with diabetes achieved NICE defined treatment targets for glucose control, blood pressure and blood cholesterol?
- What percentage of people registered with diabetes are offered and attend a structured education course?
- For people with registered diabetes what are the rates of acute and long-term complications (disease outcomes)?

Also included within the Core NDA are three other components, the:

- Diabetes Prevention Programme
- Insulin Pump report
- Diabetes Care for people with Learning Disabilities and Severe Mental Illness

**Use of data:** the NDA website sates that the data is used for:

- clinical commissioning group (CCG) diabetes profiles
- Improvement and Assessment Framework (IAF) metrics
- cardiovascular disease commissioning for value focus packs
- Diabetes Outcomes Versus Expenditure (DOVE) tool – cost effective prescribing
- Care Quality Commission (CQC) and Getting It Right First Time (GIRFT) metrics

**Participation in the audit**

General practices and specialist services are required to participate in the audit using the General Practice Extraction Service (GPES). Secondary care providers submit data manually via the Clinical Audit Platform. If individual patients have opted out of sharing their data for research and planning purposes, or “type 2 opt-out”, their data will not be used within the NDA.

**Core NDA data collection**

There is an annual audit collection for the annual report. Recently an additional quarterly data collection has been added for primary care.

**Feedback**

The core NDA audit is a data release as an Excel workbook. From that there are specialist audits which produce audit reports e.g. footcare, pregnancy in diabetes.

**Recent change to the audit**

In primary care there is now a quarterly data collection and audit release, to add to the annual report. The aim is to provide more timely data. There have been difficulties with getting this to work with the different IT systems, but a changing legal status for quarterly data means the input should get better. GP practices have to opt-in for each audit round.

**Feedback cycle**

**Are the standards of clinical performance clear?** Yes, evidence-based criteria: NICE nine processes of care.

**Who does the data collection?** Collected automatically for primary care - NHS England - mandate to collect the data. Secondary care - audit data is submitted from units - numbers are increasing as they bring in/find more units not previously included.

**What feedback is communicated?** A notification is sent to all on mailing lists - have to request to be on the mailing list. The core audit is purely a data release on an Excel workbook. Analysis is done by the specialist audit workstreams e.g. footcare.

**How is the feedback received? Is there interaction?** For primary care the target audience is “GPs/ practice business manager to manage change”. Interaction with users: the NDA deals with direct queries; Diabetes UK is subcontracted to do user contact, feedback, QI projects, summary information, clinical lead engagement. It seems that some practices do not receive any NDA feedback; some CCGs collate the data, some have dashboards that use the NDA data; highly variable

**How is the feedback understood?** “responsibility – NDA have advisory groups of e.g. specialists who steer the reports from specialist audits - analysis of interesting data found etc.” [what are the expectations to act?]

**Can the recipients interrogate the data?** Need to be able to work with Excel to look at the core data; it can take time to find your own practice code. If want to compare with previous reports, need to have kept past reports and do the comparison for your own practice data, and to compare with other practices (it is possible to make a data access request to the NDA).

**Is there acceptance of the feedback?** There is no tailoring or segmentation for the core audit (there is for the specialist audit reports).

**Does the feedback elicit a planned response?** Not known.

**Is the behavioural response at patient or organisation level?** Long term condition so behaviour is for ongoing care. NDA says reports and recommendations are aimed at different levels: CCG, practice, patient: Diabetes UK involved.

**Are there positive changes to patient care as a result of feedback?** There is a lag time in DM to see tangible changes.

**Are there any unintended consequences as a result of the feedback?** Not known.


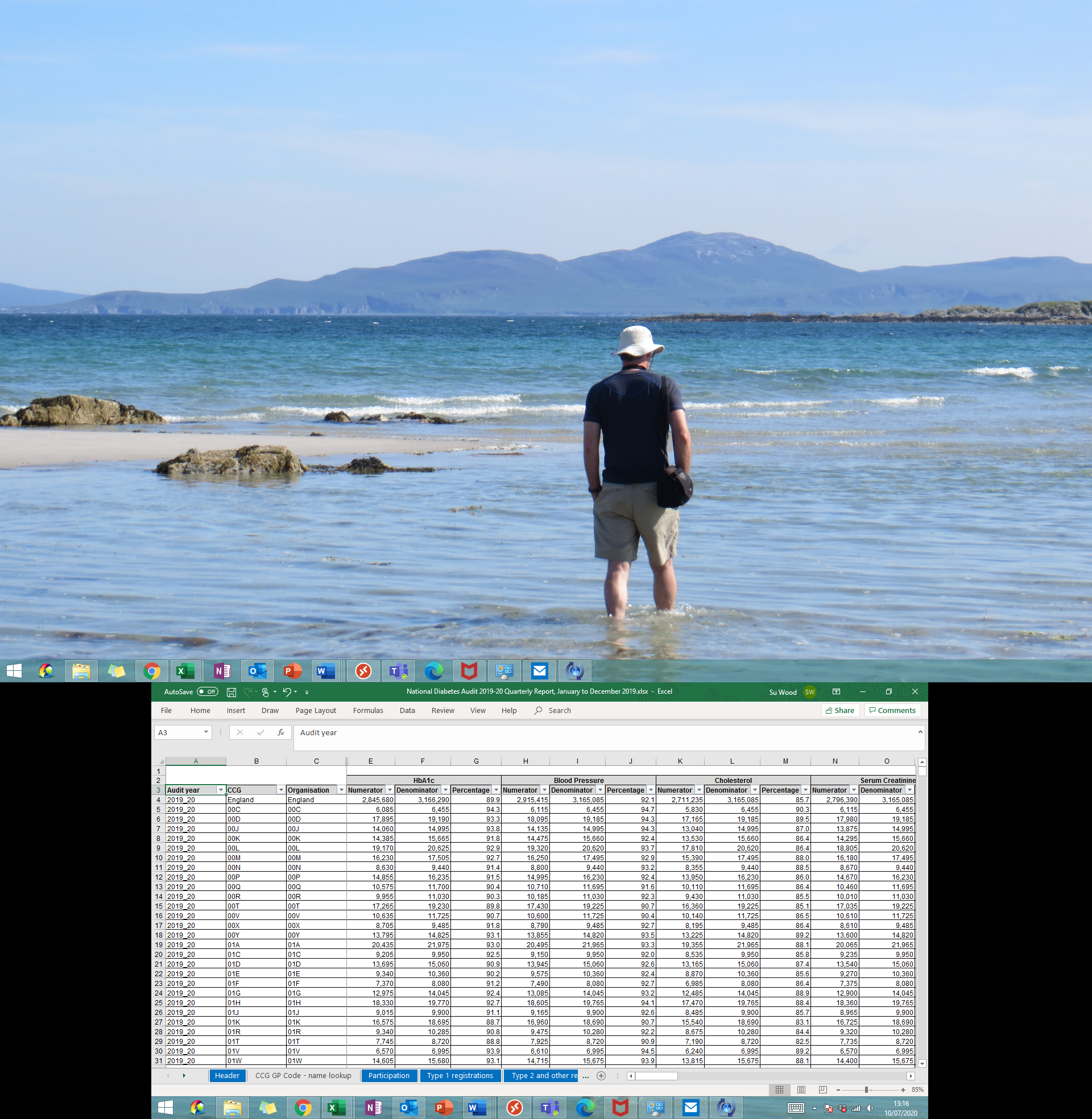
**Example core NDA service level report: screen shot**

**NDA national report – example slide**

**The Trauma Audit & Research Network (TARN) Summary**

<https://www.tarn.ac.uk/Home.aspx>

TARN is a national organisation, based at Manchester University, that collects and processes data on moderately and severely injured patients from hospitals. It allows networks, major trauma centres, trauma units, ambulance services and individual clinicians to benchmark their trauma service with other providers across the country.

TARN was established in 1990, and the committee and network was set up in 2000. The dashboard has been running since 2012 with quarterly reporting on data quality (case accreditation) and key performance measures (standards of care performance), with trends and benchmarking.

The TARN board is chaired by professor of emergency medicine, and includes people with relevant clinical skills, and PPI representatives. There are then subcommittees e.g. the audit committee of 25ish people, including clinicians, co-ordinators, managers, PPI.

Research is an established part of TARN with 100+ research projects - good cycle of research feeding back into the standards and audit e.g. head injury study led to changes in NICE and to change in standards in TARN.

**Participation in the audit**

Since 2012 all trauma units have had to submit data to TARN; they were reconfigured into trauma networks at that time. Major trauma networks comprise of a collection of service-providers and personnel, who serve a defined population and aim to reduce death and disability following injury. Each network is served by one or more Major Trauma Centres, along with a number of smaller hospitals called trauma units, and several prehospital care providers.

There is an annual subscription fee to TARN based on annual attendance, which goes to running the audit.

All Major Trauma Centres can get a ‘Best Practice Tariff’ if required elements of care are delivered – these need to be captured in the TARN data set in order for payment to be received.

**Data collection**

Individual patient data is inputted manually at the trauma unit, aiming to be within 25 days of discharge/death. TARN provides training for coders.

**Feedback**

Feedback is reported in different ways: the dashboard is updated quarterly, childrens dashboard twice a year, PROMs quarterly, clinical feedback reports three times a year with comparative data, national reports every 2 years (incl for children, older people), and ad hoc reporting on request.

**Recent change to the audit**

TARN has recently introduced the ‘TARN Analytics’ tool as an enhanced method of information access with data in ‘useful and effective visualisations’. They state that they wanted to give greater access to the front line - "it is their data". They were getting a lot of ad hoc analysis requests suggesting wanting to be able to understand the data more and dig down further. Reports are sent to clinical leads, but they are wanting to get more engagement from consultants etc.

**Feedback cycle**

**Are the standards of clinical performance clear?** Yes. Continually working on ensuring standards current; research to inform; input from consultants and networks.

**Who does the data collection?** Trauma units input individual patient data manually. Mandatory for trauma units to take part since 2012.

**What feedback is communicated?** Email notification to login holders when new data released. Dashboards; clinical feedback reports with comparative data; PROM quarterly; national reports every 2 yrs; ad hoc analysis when requested. Reports are on data quality (case accreditation) and key performance measures (standards of care performance), with trends and benchmarking.

**How is the feedback received? Is there interaction?** There is positive reaction to the feedback, and regular interaction with TARN. “TARN do listen to users, I feel involved in development.” [network manager] An example from TARN: when outliers are identified (positive and negative) they work with hospitals to identify why, starting with looking at data quality – “it is always a positive process with positive engagement” [TARN].

**How is the feedback understood?** Up to now has gone to network managers etc to analyse and present to the teams – an aim of the new Analytics Tool is to make it more understandable, accessible and useable to more on the ground - consultants etc. There is now use of Red/Amber/Green to give an immediate visual of where work is needed.

**Can the recipients interrogate the data?** Yes, and will be easier with the new Analytics Tool.

**Is there acceptance of the feedback?** Yes, “unless we don't think the data fits what we think is happening in the unit” [network manager]; data is inputted by the unit. The Analytics Tool is aimed to help users drill down to understand the data.

**Does the feedback elicit a planned response?** Yes “where low in the figures, and where there is leadership for change”.

**Is the behavioural response at patient or organisation level?** One off acute patient episodes, so each entry is different (not ongoing care). The feedback is aimed at organisation level.

**Are there positive changes to patient care as a result of feedback?** “TARN has transformed patient care.” [network manager] The TARN website has examples of the improvement in patient care over time, including patient survival.

**Are there any unintended consequences as a result of the feedback?** TARN say they try to be very careful about how the audit is implemented and be aware of the consequences. A network manager suggests if not experienced with the statistics (public/ new staff etc) then can interpret wrongly so can lead to wrong reaction to the figures e.g. when patient numbers are small; or problem of using a national average if it is low.

**TARN Dashboard example**

**TARN Analytics tool example screens**
