# Supplementary material for: Opportunities to improve the impact of two national clinical audit programmes: a theory-guided analysis
Source: Implement Sci Commun. 2022 Mar 21;3:32. doi: 10.1186/s43058-022-00275-5 (PMC8935621; doi:10.1186/s43058-022-00275-5)
Supplement: Supplementary file 3 — Additional file 3. CP-FIT feedback cycle and questions for discussion. [file 43058_2022_275_MOESM3_ESM.docx]

| **Additional File 3. CP-FIT feedback cycle and questions for discussion** | |  |
| --- | --- | --- |
|  |  |  |
| **CP-FIT components** | **Questions to ask of the audit** | **Comments** |
| Goal setting | Are the standards of clinical performance clear? |  |
| Data collection | Who does the data collection? |  |
| Feedback | What feedback is communicated? |  |
| Interaction | How is the feedback received? |  |
| Perception | How is the feedback understood? |  |
| Verification | Can the recipients interrogate the data? |  |
| Acceptance | Is there acceptance of the feedback? |  |
| Intention | Does the feedback elicit a planned response? |  |
| Behaviour | Is the behavioural response at patient or organisation level? |  |
| Clinical performance improvement | Are there positive changes to patient care as a result of feedback? |  |
| Unintended consequences | Are there any unintended consequences as a result of the feedback? |  |
