# Supplementary material for: Opportunities to improve the impact of two national clinical audit programmes: a theory-guided analysis
Source: Implement Sci Commun. 2022 Mar 21;3:32. doi: 10.1186/s43058-022-00275-5 (PMC8935621; doi:10.1186/s43058-022-00275-5)
Supplement: Supplementary file 6 — Additional file 6. Results for the remaining CP-FIT feedback cycle components. [file 43058_2022_275_MOESM6_ESM.docx]

**Additional File 6.**

**Results for the remaining CP-FIT feedback cycle components**

**National Diabetes Audit**

Goal-setting (Figure 1, feedback cycle component 1)

The NDA core audit is well-established and includes clinical indicators of recognised public health importance. This relates to two of CP-FIT’s high-confidence hypotheses, i.e. that feedback interventions are more effective when they focus on goals that recipients a) believe to be meaningful, and b) perceive as relevant to their role. The NDA’s clear, evidence-based criteria were considered a strength.

Verification

Interviewees acknowledged that the quarterly data release permitted greater interactivity. Data were presented in an Excel spreadsheet, a format likely to be familiar to recipients:

“*The advantage of Excel is that I think most GPs could open the file! Because I’ve learned that a lot of GPs are not tech-savvy, and don’t wish to be*.” D17, Audit and feedback researcher and GP

However, participants were not convinced that recipients would actually interrogate and verify the data. Interviewees with primary care experience wanted the ability to identify patients in need of review. Otherwise, it was difficult to see how the data could prompt specific actions to enable improvement.

“*There’s no point sending back data to practices if they can’t do anything about it. If they can’t run a similar search find out who they need to see and review and where there’s gaps in care, then you’re making it very difficult for people to interact and actually achieve something different*.” D17, Audit and feedback researcher and GP

Clinical performance improvement

Interviewees were generally unconvinced that feedback would improve patient care given for lack of supporting mechanisms for doing so. One GP considered the audit as supportive of positive change, contrasting with other approaches that take a more punitive response to underachievement (e.g. inspection regimes) (D16, audit and feedback researcher and GP).

Participants touched upon what CP-FIT terms “observability”: feedback interventions are more effective when their potential benefits are demonstrated to recipients. They suggested including messages to highlight the population benefits of greater adherence to recommended practice:

“*I think what they need to do, or consider, would be something like, ‘Out of your 400 people with Type 2 diabetes there are 100 with sub-optimally controlled blood pressure. If you were to improve that control for 50 of those, set a reasonable target over the next year, and maintain that improvement, that would translate into fewer strokes in the next ten…’ They can’t see what the tangible benefits are for patients just now because it’s processes*.” D2, audit and feedback researcher and GP

Unintended consequences

CP-FIT acknowledges the potential for both positive and negative unintended outcomes of feedback interventions, e.g. improved record-keeping, or manipulation of patient populations to ‘game’ the data and artificially improve performance. Our participants identified no such unintended consequences in relation to the NDA.

**TARN**

Goal-setting

Our interviewees considered that TARN’s clinical performance standards were both important and relevant to recipients’ roles, in line with CP-FIT hypotheses. One participant (D5, trauma network manager) used TARN outputs within his professional role and recognised their application to patient care.

Feedback

An interviewee working in trauma care praised the TARN feedback. He felt that TARN sought to provide accessible outputs that balanced recipients’ varying information needs. He proceeded to consider the function of feedback: to support positive change or punish poor performance?

“*I think it comes across as neutral. I think that’s nice from TARN’s perspective because they’re not meant to be choosing to manage or lead in a carrot or stick format. I think that’s a choice for managers or clinical directors or chief execs or leaders as to what balance and proportion of carrot and stick they use in their day job to make change happen*.”

Importantly, however, feedback still must stimulate change:

“*There’s a negative to that though, is that neutrality doesn’t help change anything*.” (both D5, trauma network manager)

Unintended consequences

A suggested negative consequence of TARN feedback, albeit relevant to all clinical audits, concerned how data are portrayed, particularly in relation to the public:

*“I don’t think that we write these things for patients, and so I think unintended consequences are when people pick up in data that … if patients don’t have a statistical knowledge of what the data means then they miss – it’s the Daily Mail approach isn’t it? You know, you have to have a sort of shock headline, you know, that always has to be something that’s gone wrong, rather than something that’s gone right […] I think there’s a real challenge that if you’re, if you have public –facing national audit and you don’t present it in a way that people will understand then they’ll misinterpret it, so that’s an unintended consequence*.” D19, consultant and former national audit lead
